# Hypertension and Its Associations with Dental Status: Data from the Dental, Oral, Medical Epidemiological (DOME) Nationwide Records-Based Study

**DOI:** 10.3390/jcm10020176

**Published:** 2021-01-06

**Authors:** Galit Almoznino, Avraham Zini, Ron Kedem, Noam E. Protter, Dorit Zur, Itzhak Abramovitz

**Affiliations:** 1 Big Biomedical Data Research Laboratory, Hadassah School of Dental Medicine, Hebrew University, Jerusalem 91120, Israel; 2 Department of Endodontics, Hadassah School of Dental Medicine, Hebrew University, Jerusalem 91120, Israel; itzhakab@hadassah.org.il; 3 Department of Oral Medicine, Sedation & Maxillofacial Imaging, Hadassah School of Dental Medicine, Hebrew University, Jerusalem 91120, Israel; 4 Department of Community Dentistry, Hadassah School of Dental Medicine, Hebrew University, Jerusalem 91120, Israel; aviz@hadassah.org.il; 5 Medical Information Department, General Surgeon Headquarter, Medical Corps, Israel Defense Forces, Tel-Hashomer 02149, Israel; ron.kedem56@gmail.com (R.K.); Dorit48@mail.idf.il (D.Z.); 6 Forensic Unit, Medical Corps, Israel Defense Forces, Tel-Hashomer 02149, Israel; noamprotter@gmail.com

**Keywords:** hypertension, dental, caries, decayed teeth, metabolic syndrome, diabetes mellitus, hyperlipidemia, C-reactive protein, obesity, electronic medical record, electronic dental record

## Abstract

Conflicting results have been published regarding the associations between dental status and hypertension. This study aims to explore whether or not hypertension is associated with dental status among young to middle-aged adults. To that end, data from the Dental, Oral, Medical Epidemiological (DOME) study were analyzed. The DOME is a cross-sectional records-based study that combines comprehensive socio-demographic, medical, and dental databases of a nationally representative sample of military personnel. Included were 132,529 subjects aged 18–50 years who attended the military dental clinics for one year. The prevalence of hypertension in the study population was 2.5% (3363/132,529). Following multivariate analysis, the associations between hypertension and dental parameters were lost and hypertension retained a positive association with obesity (Odds ratio (OR) = 4.2 (3.7–4.9)), diabetes mellitus (OR = 4.0 (2.9–5.7)), birth country of Western Europe vs. Israeli birth country (OR = 1.9 (1.6–2.2)), male sex (OR = 1.9 (1.6–2.2)), cardiovascular disease (OR = 1.9 (1.6–2.3)), presence of fatty liver (OR = 1.8 (1.5–2.3)), the birth country Asia vs. Israeli birth country (OR = 1.6 (1.1–2.3)), smoking (OR = 1.2 (1.05–1.4)), and older age (OR = 1.05 (1.04–1.06)). Further analysis among an age-, smoking- and sex matched sub-population (*N* = 13,452) also revealed that the dental parameters lost their statistically significant association with hypertension following multivariate analysis, and hypertension retained a positive association with diabetes (OR = 4.08 (2.6–6.1)), obesity (OR = 2.7 (2.4–3.2)), birth country of Western Europe vs. Israel (OR = 1.9 (1.6–2.3)), cardiovascular disease (OR = 1.8 (1.5–2.2)), fatty liver (OR = 1.7 (1.3–2.3)), high school education vs. academic (OR = 1.5 (1.3–1.8)), and low socio-economic status (SES) vs. high (OR = 1.4 (1.03–1.8)). We analyzed the associations between C-reactive protein (CRP) and dental parameters and combined the statistically significant variables to create a dental inflammation score (DIS). This crated a final model with the appropriate weights written as follows: DIS = (periodontal disease × 14) + (the number of teeth that required crowns × 11) + (missing teeth × 75). The mean DIS was 10.106 ± 25.184, and it exhibited a weak positive association with hypertension in the univariate analysis (OR = 1.011 (1.010–1.012)). Receiver operating characteristic (ROC) analysis of the DIS against hypertension produced a failed area under the curve (AUC) result (0.57 (0.56–0.58)). Moreover, the DIS also lost its statistical significance association with hypertension following multivariate analysis. We conclude that hypertension had no statistically significant nor clinically significant association with dental status. The study established a profile of the “patient vulnerable to hypertension”, which retained well-known risk factors for hypertension such as older age, male sex, smoking, diabetes, obesity, and fatty liver but not dental parameters.

## 1. Introduction

Hypertension is a chronic disease that is often dubbed the “silent killer” as it rarely causes symptoms, but simultaneously it is an independent risk factor for coronary heart disease, stroke, renal disease, peripheral arterial disease, and vascular cognitive impairment [1,2]. Hypertension is one of the components of metabolic syndrome (MetS), along with diabetes mellitus, hyperlipidemia, abdominal obesity, and fatty liver [3].

The global prevalence of hypertension was estimated at 1.3 billion in 2015 [4,5]. The data indicate that hypertension among young adults is more common than what was frequently thought. Among men aged 18–39, there was a higher prevalence of 15–20% in men and 12–15% in women, which was even higher among overweight people [6,7]. In addition, younger adults have significantly higher rates of under-diagnosis of hypertension [6,7], and lower rates of blood pressure control [8,9] than the rates among those aged 40 and older.

Associations between dental problems, hypertension and cardiovascular diseases (CVD) have been previously investigated. A recent consensus report updated the existing epidemiological evidence for significant associations between periodontitis and cardiovascular diseases, although a causal relationship has not been established [10]. The proposed mechanisms include bacteremia and the associated systemic inflammatory sequelae, including elevations in C-reactive protein (CRP) and oxidative stress [11]. Given that endodontic infections share several characteristics with periodontitis, including altered microbiota and pro-inflammatory mediators, their role in CVD has also been investigated [12]. Indeed, oral infections in childhood appear to be associated with the subclinical carotid atherosclerosis seen in adulthood [13]. The consensus report concluded that there is now a significant body of evidence to support independent associations between severe periodontitis and several non-communicable diseases including diabetes, cardiovascular disease, chronic obstructive pulmonary disease and chronic kidney disease, and even with all-cause and cardiovascular mortality in several populations [10]. These associations had not been established specifically in hypertension, however considering that hypertension is part of the metabolic syndrome, there is a need for large-scale epidemiological studies that will assess the inflammatory theorem in the context of hypertension, by studying the association between hypertension, dental morbidities and markers for systemic inflammation, such as CRP. Indeed, when assessing the association between dental parameters and hypertension, the studies should distinguish between cases with hypertension as a sole diagnosis, vis-à-vis cases where hypertension exists as part of the metabolic syndrome cluster.

Moreover, while some studies support the association of hypertension with caries [14,15], other studies did not find a significant association between carious teeth and hypertension [16,17]. These conflicting results could be attributed to the limitations of the published studies, such as heterogeneity in the definitions of dental and systemic diseases and the presence of possible confounders that were not always considered. For example, there are well-known common risk factors [18] for many chronic diseases, including dental caries and hypertension [19,20,21]. Among the common risks are increased age, socioeconomic status, smoking, alcohol consumption, and obesity [22,23].

Considering these limitations, there is a need for large-scale studies with a rigorous protocol regarding dental and medical disease definitions, which take into account the existence of many possible confounders. In particular, it is important to assess the associations between dental status and hypertension among young and middle-aged adults due to the relatively high prevalence of hypertension, as well as the lower rates of diagnosis in these age groups.

To address the gap in the literature, the primary objective of this study was to analyze the association of hypertension with dental status among young and middle-aged adults. To that end, the current study used data from the “The Dental, Oral, Medical Epidemiological (DOME)” study [24]. The DOME is a cross-sectional records-based study that combines comprehensive socio-demographic, medical, and dental databases of a nationally representative sample of military personnel from the Israel Defense Forces (IDF) [24]. The methods of data collection of the DOME study were described in detail previously [17,24,25,26]. An unusual opportunity exists in Israel to study these associations using this comprehensive information collected in the military databases and captured in the DOME repository. The military population in Israel is large and comprises a credible data source for epidemiological studies among young and middle-aged adults. This is partly since conscription exists in Israel for all Jewish, Druze, or Circassian citizens over the age of 18 [17]. Importantly, service in the IDF includes medically complex individuals, except for those who are unfit for service for health reasons (physical or mental), and even for those subjects, there is an option to apply for volunteering [27,28]. Dental services are part of the comprehensive medical care, and the IDF military personnel do not incur any medical and/or dental expenses [29,30].

In our recent publications, we coined the term “SOS teeth” to represent teeth that need to be treated first due to advanced caries reaching the pulp or the presence of decayed root fragments [17,26]. Using the DOME database, we have previously shown that teeth with advanced caries termed “SOS teeth” had no statistically significant association with metabolic syndrome [17]. In this study, we focused on hypertension and further explored its association with dental treatment needs and actual dental treatments. Specifically, we assessed the associations of hypertension with the prevalence of the following dental treatment needs and actual dental procedures: (1) fillings, (2) endodontic treatments, (3) post fabrications, (4) crowns, (5) extractions, (6) periodontal disease and (7) missing teeth. Assessment of other dental parameters beyond these dental parameters is beyond the scope of this paper. To assess possible confounders, we also analyzed the associations of hypertension with socio-demographic parameters, health-related risk habits, medical and dental attendance patterns, and medical diagnoses of the subject. This will enable us to establish a profile of the “patient vulnerable to hypertension”, and to see if following the multivariate analysis, the profile will still include dental parameters. Furthermore, in order to assess the inflammatory theorem presented above, another objective of this study was to analyze the associations between CRP and the dental conditions, combine the statistically significant variables to create a dental inflammation score (DIS), assign weights according to their importance, and use the DIS as a predictor for hypertension.

The main research hypothesis is that poor dental status, reflected by a higher prevalence of dental treatment needs, will be the predictor of the outcome of hypertension. Nevertheless, we hypothesized that these associations and correlations may be explained by shared risk factors, and therefore will be lost after controlling for socio-demographics, common health-related habits, and other systemic morbidities. This hypothesis was based on our prior evidence when analyzing the associations between metabolic syndrome and periodontal disease, as well as SOS teeth [17,31].

## 2. Methods

### 2.1. Study Population

The study included data from the DOME study [24], which captures the socio-demographic, medical and dental records of all IDF military personnel, over the age of 18 years and of both sexes, who attended military dental clinics of the Israel Defense Forces (IDF) between January 1st, 2015 and January 1st, 2016, for which there are records in the DPR (dental patient Record) database [24]. Excluded were subjects with an absence of data in these databases.

### 2.2. Data Collection

A full description of the data collection, protocols, and methods of the DOME study had been previously published [24]. In brief, the DOME is a structured repository that captures three military electronic databases [17,26]: (a) DPR, dental patient record—this electronic dental record (EDR) system stores the dental records of all military dental attendees; (b) MPR, medical patient record—this is a comprehensive electronic medical record (EMR) system that stores the general medical records of all military personnel [32]; (c) the IDF’s central demographic database keeps the personal socio-demographic profiles of the military population [24,33].

### 2.3. Study Variables

We analyzed the associations and correlations between hypertension as a dependent variable and independent variables. Definitions of the variables available in the DOME repository have been detailed in the DOME protocol and methods paper previously [24], and will be described briefly below.

#### 2.3.1. Definitions of Dental Parameters

Standardized codes for dental procedures in the dental patient record (DPR). Standardized uniform codes are employed in the DPR for each dental procedure [24]. As described in the protocol of the DOME [24], the DOME repository includes uniform codes for dental procedures employed in the DPR that are equivalent to the nomenclature used by the American Dental Association’s (ADA) Current Dental Terminology (CDT) [34]. Each dental code was drawn from the computerized database twice: once as the treatment plan needs value (assigned as “required”), and secondly as the value of the treatments that were actually performed for the specific parameter (assigned as “performed”). Definitions of the included dental treatment needs and actual dental procedures and their equivalent ADA-CDT codes are as follows:

(1) Fillings—(a) Amalgam, one surface, permanent (D2140). (b) Amalgam, two surfaces (D2150). (c) Amalgam, three surfaces and more (D2160). (d) Amalgam crown (D2161). (e) Resin-based composite fillings (D2330, D2331, D2332, D2335). The total number of fillings was the sum of these codes [24,34];
(2) Endodontic treatments—(a) Endodontic therapy, one root canal (D3310). (b) Endodontic therapy, two root canals (D3320). (c) Endodontic therapy, three or more root canals (D3330). In the present study we included the total number of endodontic treatments which was the sum of these codes [24,34];
(3) Direct post fabrications—prefabricated post and core in addition to a crown (D2954) [24,34];
(4) Crowns—(a) Crown, porcelain/ceramic (D2740). (b) Crown, porcelain fused to high noble metal (D2750). In the present study we included the total number of crowns which was the sum of these codes [24,34];
(5) Extractions—Extraction erupted tooth or exposed root (D7140) [24,34].

In addition to procedures, the DPR data include records of the presence of periodontal disease and a count of missing teeth for any reason (excluding wisdom teeth) [24]. The standardization process as well as definitions of standardized diagnostic criteria for all dental parameters were described in detail previously in the DOME methods paper [24]. In brief, examinations were performed according to the guidelines of the Dental Department of the IDF medical corps, in an indoor setting, using a pair of vertical bilateral bitewings for the molar and premolar areas for all subjects, and periapical radiographs for deep caries, endodontically treated teeth, and periodontal disease [24]. To ensure standardization administrative as well as clinical workup, all military dentists complete routine training wherein the guidelines and protocols are reviewed, and there are regular quality assessment (QA) audits [24].

#### 2.3.2. Definitions and Measurement of the Socio-Demographic Predictors

The definitions and measurement of the socio-demographic parameters were described in detail previously in the DOME methods paper [24]. In brief, the socio-demographic variables drawn from the IDF’s central demographic database included the following [24]: age, sex (men/women), service duration (months), education (high school/technical college/academic), locality of residence (urban Jewish, urban non-Jewish, rural), socio-economic status (SES) (drawn from the records of the Israeli Ministry of the Interior (low (1st–4th deciles), medium (5th–7th), and high (8th–10th)), rings of a city/town (midtown/suburbs), and countries of birth [33,35,36] (Western Europe, Former Soviet Union (FSU), Asia, Ethiopia, Africa, North America, South America, Israel) [24].

#### 2.3.3. Definitions of Health-Related Habits

Current smoking and alcohol as depicted in the MPR records, based on self-reported consumption status (yes/no) [17,24].

Health-related habits derived from the DPR included assessment of the following (yes/no): (a) Teeth brushing at least once a day; (b) Consumption of cariogenic diet (the use of snacks and/or sweets between meals or instead of meals); (c) Sweetened beverages (exposure to sweet drinks over one cup a day) [24].

#### 2.3.4. Definitions of Medical and Dental Attendance Patterns

Assessment of health care utilization during the study period included the total number of appointments with a general physician, the total number with a dentist, and the number of non-attendance to scheduled dental appointments [24].

#### 2.3.5. Definitions of General Health Status Parameters

The general health status parameters that were obtained from the MPR included the following medical diagnoses, as was detailed previously [17,24]:

(1) The dependent variable—hypertension diagnosis;
(2) Other systemic conditions related to metabolic syndrome were included as independent variables—hyperlipidemia, diabetes mellitus, impaired glucose tolerance (IGT), obesity, cardiovascular disease, fatty liver, obstructive sleep apnea (OSA), anemia, and C-reactive protein levels [17,24].

### 2.4. Statistical Methods

Data were tabulated, and statistical analyses were performed using SPSS software version 25.0 (IBM, Chicago, IL, USA).

#### 2.4.1. Descriptive Statistics

Continuous variables are presented as means and standard deviations. Categorical variables are presented as frequencies and percentages.

#### 2.4.2. Explanatory Statistics—Univariate Analysis

The associations between hypertension and independent variables were examined with Pearson Chi-Square test or likelihood ratio test (for categorical parameters), and with a non-paired t-test for independent samples (for continuous variables). The assessment of normal distributions of all the continuous parameters was performed and revealed a lack of normal distribution. Therefore, we also used the non-parametric Mann–Whitney test, which does not require the assumption of normal distributions to analyze the association between hypertension diagnosis and the independent continuous variables. Due to the large sample size, and since there were no differences in the statistical significance between the t-test results and the Mann–Whitney test results, we present the t-test results for all the correlations between hypertension diagnosis and the independent continuous parameters in this study.

#### 2.4.3. Calculations of Odds Ratios (OR)

OR were calculated for the continuous variables using linear regression analysis, and for categorical variables, the calculation was performed using binary logistic regression analysis.

#### 2.4.4. Addressing the Large Sample Size and Possible Confounders

Following the univariate analyses, a multivariate analysis was performed using multivariate logistic regression analysis for hypertension diagnosis as the dependent variable. The criteria for independent variables to enter the multivariate analysis were as follows:

(1) A statistically significant association with hypertension diagnosis in the univariate analysis. Due to the large sample size, a *p* value of <0.01 (2-tailed) in the univariate analysis was considered statistically significant to enter the multivariate analysis;
(2) Multicollinearity tests—before entering a statistically significant variable in the univariate analysis into the multivariate model, the collinearity of the independent variables was examined. If two or more variables were found highly collinear, only one of them was included in the model, and it was decided by the context which of the variables will be included in the analysis. A *p* value of <0.01 level (2-tailed) was also considered significant for the multicollinearity tests. The multicollinearity tests between the variables comprising the DOME repository have been previously published [24]. A linear regression analysis was performed to assess collinearity between the independent variables. The variance inflation factors (VIFs), which are 1/Tolerance, are presented in the linear regression analysis. The results ruled out collinearity (VIF < 2.5). Although values of VIF below 10 are usually regarded as indicating multicollinearity, in weaker models values above 2.5 may be a cause for concern. Therefore, the cutoff of VIF in the present study was 2.5.
(3) Multivariate analysis—Finally, the multivariate logistic regression analysis was used as a statistical model, by including in the analysis simultaneously all the variables fulfilling the criteria as described above. Due to the large sample size, a *p* value of <0.01 (2-tailed) was also considered statistically significant in the multivariate analysis.

## 3. Results

### 3.1. Description of the Study Population and the Prevalence of Hypertension

The study included 132,529 records of patients who met the eligibility criteria of the study. The mean age of the whole study population 21.88 ± 6.02 years, the age range was 18–50 and the median age was 20 years. The prevalence of hypertension in the study population was 2.5% (3363/132,529). Among the study population, the mean number of decayed teeth was 2.10 ± 2.8 and the mean number of missing teeth was 0.58 ± 1.3. Men compared to women exhibited higher mean number of both decayed (2.1 ± 2.8 vs. 1.9 ± 2.6, *p* < 0.001) and missing teeth (0.6 ± 1.3 vs. 0.52 ± 1.1, *p* < 0.001). The number of filling teeth is not included in the DOME repository.

All analyses were performed twice:

(1) Comparison to those without hypertension using the whole study population (*N* = 132,529 subjects; of those, 3363 with hypertension and 129,166 without hypertension) (presented on the left side of the Tables);
(2) Comparison to age-, sex- and smoking-matched group without hypertension, which is three times larger than the hypertension group (*N* = 13,452 subjects: of those, 3363 with hypertension and 10,089 without hypertension) (presented on the right side of the Tables).

To create the age-, sex- and smoking-matched group, we stratified the data according to sex (male/female), smoking (smoker/no smoker) and age, the latter of which was dichotomized into younger (<30 years) (the ~mean age of the hypertension group) and older (≥30 years). This resulted in eight possible strata, as can be seen in Table A1 in the appendix. We created a “control group without hypertension” that was three times larger than the hypertension group, according to the weight on each strata. As can be seen in Table A1, the likelihood ratio analysis of the “Strata” variable against “hypertension” revealed *p* = 1.0.

### 3.2. The Associations between Socio-Demographic Parameters and Hypertension

Table 1 presents the associations between socio-demographic parameters and hypertension among the study population.

Comparison to those without hypertension among the whole study population: Compared to those without hypertension, hypertension was statistically significantly positively associated with male sex (OR men/women 2.8 (2.5–3.2)), technical education (OR technical/academics 1.3 (1.2–1.5)), medium vs. high SES (OR for medium/high 1.1 (1.0–1.2)), living in the peripheral rings of a city/town (OR periphery/central city rings 1.2 (1.1–1.3)), age (OR = 1.1 (1.1–1.1)), time in service (OR = 1.1 (1.1–1.1)), and the following countries of birth compared to native Israelis (from the highest to the lowest OR): Asia (OR = 2.3 (1.6–3.3)), Africa (OR = 2.2 (1.4–3.6)), and Western Europe (OR = 1.9 (1.8–2.2)) (see Table 1).

Compared to those without hypertension, the hypertension group had a statistically significant negative association (i.e., had a lower prevalence of hypertension) with the following variables: Urban Jewish (OR = 0.3 (0.2–0.4)) and Urban non-Jewish (OR = 0.2 (0.1–0.3)) localities compared to the rural locality, immigrants from Ethiopia (OR = 0.5 (0.4–0.8)) and North Americaa (OR = 0.5 (0.3–0.7)) birth countries (see Table 1).

The comparison to the age-, sex- and smoking-matched group without hypertension revealed similar trends except for age, sex and time in service, which were matched.

### 3.3. The Associations of Hypertension with Patient Health-Related Habits, and Attendance Patterns

Table 2 presents the associations of hypertension diagnosis with patient health-related habits, as well as the medical and dental attendance patterns.

*The health-related habits of the patient*. Hypertension was positively associated with smoking (OR = 5.6 (5.1–6.1)), alcohol consumption (OR = 3.6 (2.0–6.3)) and with brushing teeth less than once a day (OR = 1.5 (1.4–1.7)) (Table 2). Hypertension was inversely associated with the consumption of a cariogenic diet (OR = 0.7 (0.6–0.8)) and the consumption of sweetened beverages (OR = 0.8 (0.7–0.9)) (Table 2).

*Attendance patterns.* Those with hypertension had more appointments with a general physician (OR = 1.027 (1.024–1.029), had more dental appointments (OR = 1.025 (1.023–1.027)), and were more likely to not attend to scheduled dental appointments (OR = 1.060 (1.052–1.068)) (Table 2).

The comparison to the age-, sex- and smoking-matched group without hypertension revealed similar trends regarding attendance patterns; however, health-related habits were not statistically significant, except for alcohol consumption.

### 3.4. General Health Status Parameters and Periodontal Disease According to Hypertension Diagnosis

Table 3 presents the general health status parameters and periodontal disease according to hypertension diagnosis among the study population. Hypertension was positively associated with the following conditions: hyperlipidemia (OR = 6.1 (5.0–7.3)), diabetes mellitus [OR = 27.6 (22.2–34.4)], impaired glucose tolerance (OR = 6.3 (3.8–10.4)), obesity (OR = 12.7 (11.8–13.6)), cardiovascular disease (OR = 6.0 (5.4–6.7)), fatty liver (OR = 14.5 (12.5–16.9)), obstructive sleep apnea (OR = 8.7 (6.5–11.6)), anemia (OR = 1.7 (1.5–1.9)) and periodontal disease (OR = 1.4 (1.2–1.6)) (Table 3). Osteoporosis, Stroke, TIA (transient ischemic attack), DVT (deep vein thrombosis) and PVD (peripheral vascular disease) each had an extremely low prevalence among the hypertensive group. Therefore, we do not present in the table the results of their analyses against hypertension as a dependent variable, since these can be misleading.

The comparison to the age-, sex- and smoking-matched group without hypertension revealed similar trends, except for IGT, anemia and periodontal disease, which did not have a statistically significant association with hypertension in the sub-population analysis.

### 3.5. Dental Status According to Hypertension Diagnosis

Table 4 presents the requirements and performance of dental procedures according to hypertension diagnosis among the study population. Hypertension diagnosis had a weak statistically significant negative association with the following: the number of teeth that required one surface amalgam filling (OR = 0.9 (0.9–0.9)), the number of teeth where one surface amalgam filling was performed (OR = 0.9 (0.9–1.0)), and the number of teeth that required amalgam fillings on two surfaces (OR = 0.9 (0.8–0.9)).

There was no statistically significant association between hypertension and the number of teeth on which one surface amalgam filling was performed (0.9 (0.9–1.0)).

All other requirements, and the performance of the dental procedures that are presented in Table 4, had weak statistically significant positive associations with a hypertension diagnosis.

In the comparison to the age-, sex- and smoking-matched group without hypertension, hypertension had a weak positive association with the number of teeth that required one surface amalgam filling (OR = 1.043 (1.008–1.079), the total number of teeth that required fillings (OR = 1.021 (1.004–1.039)), the total number of teeth that required endodontic treatment (OR = 1.113 (1.017–1.219)), the total number of teeth whereon endodontic treatment was performed (OR = 1.112 (1.019–1.214)), the number of teeth whereon prefabricated (direct, post and core) was performed (OR = 1.117 (1.048–1.191)), the number of teeth whereon extractions were performed (OR = 1.084 (1.023–1.150)), and missing teeth (OR = 1.043 (1.009–1.078)).

### 3.6. Multivariate Analysis

#### 3.6.1. Multivariate Analysis of the Whole Study Population (*N* = 132,529)

Following the univariate analyses, a linear regression analysis was performed to assess the collinearity between the independent variables among the whole study population (*N* = 132,529) (Table 5). The results ruled out collinearity (VIF < 2.5). Following this, a multivariate logistic regression analysis was performed for hypertension diagnosis as the dependent variable (Table 5).

The parameters were entered simultaneously into the analysis. The parameters that retained a statistically positive association with hypertension in the multivariate analysis presented in Table 5 were (from the highest to the lowest OR) as follows: obesity (OR = 4.2 (3.7 −4.9)), the presence of diabetes mellitus (OR = 4.0 (2.9–5.7)), birth country of Western Europe vs. Israeli birth country (OR = 1. 9 (1.6–2.2)), male sex (OR = 1.9 (1.6–2.2)), cardiovascular disease (OR = 1.9 (1.6–2.3)), presence of fatty liver (OR = 1.8 (1.5–2.3)), the birth country Asia vs. Israeli birth country (OR = 1.6 (1.1–2.3)), smoking (OR = 1.2 (1.05–1.4)), and older age (OR = 1.05 (1.04–1.06)) (Table 5).

#### 3.6.2. Multivariate Analysis among the Age-, Sex- and Smoking-Matched Subjects

Following the univariate analyses, a linear regression analysis was performed to assess the collinearity among the sub-population of age-, sex- and smoking-matched subjects (*N* = 13,452) (Table 6). The results ruled out collinearity (VIF < 2.5). Following this, a multivariate logistic regression analysis was performed for hypertension diagnosis as the dependent variable in this sub-population (Table 6).

The parameters were entered simultaneously into the analysis. The parameters that retained a statistically positive association with hypertension in the multivariate analysis of the age-, sex- and smoking-matched sub-population that are presented in Table 6 were (from the highest to the lowest OR) as follows: the presence of diabetes mellitus (OR = 4.08 (2.6–6.1)), obesity (OR = 2.7 (2.4–3.2)), birth country of Western Europe vs. Israeli birth country (OR = 1.9 (1.6–2.3)), cardiovascular disease (OR = 1.8 (1.5–2.2)), presence of fatty liver [OR = 1.7 (1.3–2.3)], high school education vs. academic (OR = 1.5 (1.3–1.8)), and low SES vs. high (OR = 1.4 (1.03–1.8)) (Table 6).

#### 3.6.3. Dental Inflammation Score

To build a systemic inflammation theorem and explore how oral infection can contribute to it, we created a “dental inflammation score” (DIS) using CRP as a dependent variable. We used a method similar to the method employed by Janket et al. for the creation of the “asymptotic dental score” [37]. The CRP test was performed in 30,418 subjects from the total population (of those, 1387 were subjects with hypertension and 29,031 were subjects without hypertension), and the mean CRP test result was 3.7 ± 10.1 mg/L, the median was 1.1 mg/L, and the mode was 0.4 mg/L. Patients with hypertension exhibited statistically significantly higher CRP levels compared to those without hypertension (5.1 ± 11.7 vs. 3.7 ± 10.1, *p* < 0.001, OR = 1.009 (1.005–1.012)).

In the first step to create the “dental inflammation score”, we used a general linear model (GLM) to analyze the association between CRP level as the dependent variable and all the dental variables as predictors, and we chose only significant variables (Table 7). We used the likelihood ratio Chi-square from the Omnibus test results to determine the weight of each statistically significant variable. The only dental variables that had a statistically significant association with CRP were periodontal disease (likelihood ratio Chi-square: 6.77), the number of teeth that required crowns (likelihood ratio Chi-square: 5.24), and missing teeth (likelihood ratio Chi-square: 36.91) (see Table 7). We combined the likelihood ratio scores of the statistically significant variables (48.93), and for each variable, we computed the weight of this variable in the score as the likelihood ratio, then determined the weight of each variable as the likelihood ratio of each variable out of the total × 100 (Table 7). The DIS was calculated by summing these values. This created the final model with the appropriate weights, written as follows: Dental inflammation score (DIS) = (periodontal disease × 14) + (the number of teeth that required crowns × 11) + (missing teeth × 75). Each of the variables were entered into this model as dichotomized variables (presence of the dental condition: yes/no). The mean dental inflammation score (DIS) was 10.106 ± 25.184, median = 0.0, minimum = 0, maximum = 100. The hypertension group had a statistically significantly higher DIS compared to those without hypertension (19.13 ± 32.57 vs. 9.87 ± 24.91; *p* < 0.001; GLM: OR = 1.011 (1.010–1.012)).

**Figure 1:**
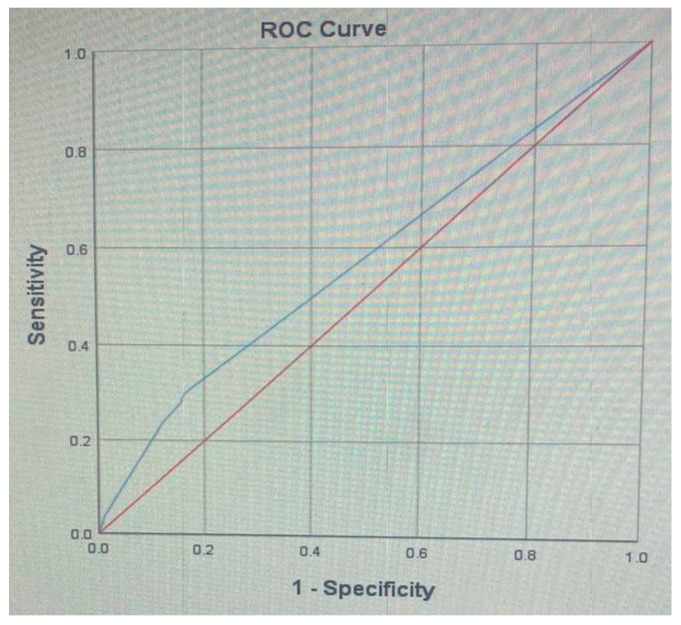
A receiver operating characteristic (ROC) analysis of the dental inflammation score (DIS) as predictor of hypertension: Area Under the Curve (AUC): 0.57 (0.56–0.58).

A receiver operating characteristic (ROC) analysis was performed on the DIS as the predictor of hypertension (Figure 1), and the area under the curve (AUC) was 0.57 (0.56–0.58), which is a fail result.

Finally, we ran a multivariate regression analysis with hypertension diagnosis as a dependent variable and all the statistically significant study variables, but instead of using the statistically significant dental variables we included the DIS. The multivariate analysis was performed among the whole study population as well as among the age-, sex- and smoking-matched sub-population. In both multivariate analyses the DIS lost its statistically significant association with hypertension, and the variables which retained statistically significant associations with hypertension were as follows:

(1) *Multivariate analysis among the whole study population*: The parameters that retained statistical significance with hypertension were, from the highest to the lowest OR, obesity (OR = 4.5 (4.06–5.0)), cardiovascular disease (OR = 1. 8 (1.6–2.1)), birth country of Western Europe vs. Israeli birth country (OR = 1. 7 (1.5–1.9)), sex (OR = 1.6 (1.5–1.8)), hyperlipidemia (OR = 1. 4 (1.1–1.7)) and age (OR = 1.04 (1.03–1.05)). The DIS lost its statically significant association with hypertension (OR = 1.0 (1.0–1.0));
(2) *Multivariate analysis among the age-, sex- and smoking-matched sub population*: The parameters that retained statistical significance with hypertension were, from the highest to the lowest OR, diabetes (OR = 4.3 (3.1–6.0)), obesity (OR = 2.7 (2.4–3.0)), fatty liver (OR = 1. 6 (1.3–2.0)), high school vs. academic education (OR = 1. 4 (1.3–1.6)), low SES vs. high (OR = 1. 3(1.1–1.6)), and technician vs. academic education (OR = 1.2(1.08–1.4)). The DIS lost its statically significant association with hypertension (OR = 1.0 (0.99–1.0)).

## 4. Discussion

The present study demonstrated that hypertension had no statistically significant or clinically significant association with dental status. Data were analyzed twice, among the whole study population as well as among an age-, sex- and smoking-matched sub-group, and in both analyses the dental parameters lost their statistically significant association with hypertension following multivariate analysis, which reflects a consistent trend. We computed a dental inflammation score (DIS) based on dental variables that had a statistically significant association with CRP. Although the DIS had a statistically significant association with hypertension in the univariate analysis, the DIS also lost its statistical significance association with hypertension following multivariate analysis. Moreover, the ROC analysis of the DIS against hypertension produced a fail AUC result (0.57). This records-based study assesses the association between hypertension and dental morbidities, combining comprehensive socio-demographic, dental and medical databases, which include dental treatment needs and actual procedures performed, socio-demographic data, medical diagnoses, health-related habits, and attendance patterns. This allowed the analysis of many confounders and mediators at the same time that were not considered concomitant previously in the medical and dental literature. The study established a profile of the “patient vulnerable to hypertension”, which retained well-known risk factors for hypertension such as older age, male sex, immigrant status, low education and low socio-economic status, smoking, diabetes, cardiovascular disease, obesity and fatty liver, but not dental parameters.

### 4.1. Dental Status and Hypertension

The associations between hypertension and dental parameters found in the univariate analysis should be interpreted with utmost caution. This is due to the large sample size, which enables us to detect even weak statistical associations that may be clinically insignificant. Indeed, in the univariate analyses, the odds ratios for some of the parameters were close to 1, suggesting a weak statistical association (see Table 4). Moreover, although the differences regarding the dental parameters between those with and without hypertension were statistically significant in the univariate analysis, the differences in the mean scores were small and clinically non-significant. For this reason, data were analyzed also among an age-, sex- and smoking-matched sub-population. The analysis of the sub-population not only reduced the huge sample size, but also controlled for important well-known confounders of age, sex and smoking. Following multivariate analysis, these weak statistical associations between hypertension and the dental parameters were lost both in the analyses of the whole population as well as among the age-, sex- and smoking-matched sub-population. These consistent results in both analyses lead us to conclude that hypertension had no statistically significant or clinically significant association with dental status.

Separately, both hypertension and dental parameters were positively associated with CRP; however, the present study could not establish a systemic inflammation theorem wherein oral infection contributes to hypertension, due to the failure to establish an association between CRP, hypertension and dental parameters, using the DIS. This could result from the weak associations between the dental parameters and hypertension. It should also be kept in mind that although patients with hypertension and with the dental pathologies comprising the DIS exhibited significantly higher CRP levels, CRP tests are not routinely performed to monitor either dental pathologies or hypertension, and the cross-sectional study design cannot address the causality between these parameters.

Instead of the systemic inflammation theorem, the findings of the present study are in line with the common risk factors approach, stating that many chronic conditions share common risk factors, and therefore changing a few risk factors can positively impact numerous diseases [18]. Genetic and environmental factors such as increased age, smoking, and lifestyle are well-known major risk factors for many chronic diseases, including metabolic syndrome, hypertension, hyperlipidemia, diabetes type 2, as well as cardiovascular disease [19,20,21], and have also been related to oral health status [18,38,39]. This common risk factors approach is in line with the WHO Global policy for the improvement of oral health in the 21st century, which concluded that because health risks are linked, preventable, and related to lifestyle, oral and general health promotion should be integrated [40]. In line with our findings, other authors also demonstrated that the significant association between edentulousness and hypertension became non-significant and was attenuated after adjustment of the potential confounders [41].

Interestingly, another possible explanation of the results could be the lower perception of pain in spontaneous or experimentally induced high blood pressure, termed “blood pressure-related hypoalgesia” [42]. The phenomenon was first described in Israel [43,44]. Since the first publication, it has been further described in the literature in many other research papers [45,46,47,48,49,50,51]. A possible hypothesis is that higher pain thresholds could account for higher rates of non-attendance to visits in the dental office, which consequently leads to more teeth with advanced carious lesions. Indeed, in the present study, patients with hypertension had a higher mean number of non-attendances to scheduled dental appointments, and had slightly more dental treatment needs, which supports this hypothesis. Unfortunately, measurements of pain thresholds were not included in this study, which excludes our ability to test this hypothesis. It is recommended that future studies will test this hypothesis by including pain thresholds as co-variants.

### 4.2. Patient Profile Positively Associated with a Hypertension Diagnosis

The study established a profile of the “patient vulnerable to hypertension”, which retained well-known risk factors for hypertension that include age, sex, birth country of Western Europe vs. native Israeli, technical education vs. academic education, the presence of diabetes mellitus, obesity, and the presence of fatty liver.

### 4.3. Socio-Demographicparameters and Hypertension

In line with our findings, previous studies have demonstrated that blood pressure progressively increases with age [52], and is more common in men [53]. Although in recent years there has been an increase in the prevalence of hypertension across all demographics, there were greater increases among men compared with women [53]. However, the prevalence of hypertension remains lower in women until menopause [54], and we can attribute the higher prevalence of hypertension among men in the present study to the inclusion of subjects aged 18–50.

The lower SES and education found in patients with hypertension in the current study is in line with the results of a meta-analysis that demonstrated that low SES is associated with higher blood pressure, and this association is particularly evident in those with a lower level of education [55]. Moreover, our finding of a positive association between hypertension and rural locality is in line with that of others, who also demonstrated that hypertension prevalence, detection, and medication use among rural adults are all significantly lower than among urban adults [56].

Regarding birth countries, the present results show that some immigrant populations have a higher prevalence of hypertension compared to native Israelis, and according to the multivariate analysis, particularly those from Western Europe and Asia. Interestingly, the 2010–2016 US National Health Interview Survey (NHIS) demonstrated that immigrants from Russia and Southeast Asia had the highest hypertension prevalence [57]. In particular, a high salt intake among Asian-born persons such as the Chinese population may make them prone to hypertension [58]. Understanding the distribution of hypertension risk factors among immigrants is an essential area in epidemiology because of current trends in migration, particularly in Israel, which is an immigrant state. Previous epidemiological studies have shown that immigrants to the US typically have better health when they arrive from their home countries than the US-born population; however, this advantage is lost with increasing years of residence in the US [59]. This phenomenon is referred to as the “healthy immigrant effect”, and is attributed to changes in the socio-economic, physical, and cultural environment [60]. The healthy immigrant effect, in the context of cardiovascular health and hypertension, may differ for particular immigrant groups, and the apparent advantage may be short-lived [57], which may explain our results. Future longitudinal studies that combine genetic and environmental assessments should further explore these observational findings.

### 4.4. Health-Related Habits and Hypertension

In the present study, smoking retained a positive association with hypertension even following multivariate analysis. Smoking is a common risk factor for both hypertension and dental/oral diseases [18]. Indeed, tobacco use is a leading cause of preventable death globally [61]. Shared risk factors, such as smoking, may account for the associations found in the univariate analysis between hypertension and dental parameters. This emphasizes the need for an appropriate risk factors management approach that should be adopted by both dental and general health clinicians, in order to control high-risk behaviors, such as smoking. While current smoking was assessed in this study, a history of past smoking was not included. Past smokers are at a higher risk of cardiovascular disease and hypertension, and relative to never-smokers, their cardiovascular risk remained significantly elevated beyond 5 years after smoking cessation [62]. Therefore, past smoking should be taken into account as a co-variant in future studies.

A sugar-sweetened diet and carbonated beverages are also risk factors for both dental caries and systemic morbidity, including hypertension [63]. In the current study, hypertension was negatively associated with cariogenic diet and with sweetened beverages consumption among the whole study population, and had no statistically significant association with hypertension in the age-, sex- and smoking-matched group. This could be attributed to the nutritional restrictions that patients with hypertension must follow. The interpretation of these parameters should be done with the utmost caution, considering that these are the only self-reported parameters in this records-based study and may be underreported.

### 4.5. The Association of Hypertension with the Systemic Morbidity of the Subject

Regarding the systemic morbidity of the subject, we found a positive association between hypertension and diseases comprising metabolic syndromes (i.e., diabetes, fatty liver, obesity), which were independently associated with hypertension. This is to be expected considering that hypertension is a part of the metabolic syndrome. Indeed, previous studies in the adult population with hypertension demonstrated higher rates of a positive parental history of hypertension [64]. The association between the triad of diabetes, fatty liver and obesity was highlighted in a recent study that showed that weight gain and the re-emergence of diabetes were associated with an increase in liver-derived plasma triglyceride, the re-accumulation of fat within the pancreas, and the recurrence of beta cell dysfunction [65]. Medical societies now recognize obesity as a chronic progressive disease [66,67], and “Diabesity” is considered a world epidemic. Not all studies that assessed the association between dental parameters and hypertension distinguished between cases with hypertension as a sole diagnosis and cases wherein hypertension exists as part of the metabolic syndrome cluster. Dental parameters may be associated with diseases comprising the metabolic cluster other than hypertension. Therefore, adjustment for other diseases comprising the metabolic syndrome is crucial so as to understand the association between hypertension and dental parameters.

Anemia was positively associated with hypertension in the univariate analysis of the whole study population, but not among the age-, sex- and smoking-matched sub-population. This may reflect the differences in hemoglobin levels across age, sex and smoking that were lost following matching. Another interesting explanation for the association between hypertension and anemia can be attributed to anemia of inflammation, also known as anemia of chronic disease, which is prevalent in patients with diseases that cause prolonged immune activation, including infection, autoimmune diseases, and cancer [68]. More recently, the list has grown to include aging, obesity, diabetes mellitus and pulmonary arterial hypertension, chronic liver disease, and advanced atherosclerosis, with its sequelae of coronary artery disease and stroke [68].

The lack of adjustment for these important parameters leads to a failure to address the complexity of the clinical scenario, and it may explain why previous studies reported on epidemiological associations between hypertension and dental problems, which might have been lost following multivariate analysis.

### 4.6. Strengths and Limitations of the Study

The main strengths of the present study are the large sample size (132,529 records) and the strict protocol for utilizing the comprehensive socio-demographic, medical, and dental databases as part of a structured study termed the DOME study [24]. Since Israel is an immigrant country, multiple ethnicities were included, which may enhance the generalizability of the results. The IDF socio-demographic, medical, and dental systems are homogenous, with standardized uniform administrative, medical and dental clinical workup and training programs for dentists and physicians regarding diagnostic and treatment protocols, as well as the uniform computerized codes. Dental examinations in the IDF are accessible and free. However, there are cases of refusal, missed examinations, or treatments in civilian dental clinics, which might cause under-documentation.

Many possible confounders were taken into account; however, due to the complexities of the topic, and the limited ability of one paper to address all possible parameters, there are other contributing factors which were not included, such as medications use, physical activity, level of control of hypertension (systemic parameters), as well as the assessment of periapical area, reasons and diagnoses for endodontic treatments and extractions, and specific numbers of teeth (dental parameters). Future studies should assess these parameters. Although most parameters are records-based and were coded by authorized professionals (socio-demographics, dental, and medical data), there are some self-reported parameters, such as health-related behaviors, which are reported by the patients, and are therefore subjected to recall bias. Due to the cross-sectional study design, we cannot assume causality, and therefore this study can only address associations and correlations between the variables. The limitations of the study include the convenience sample, which may limit the generalizability of the results. Additional studies, including long-term longitudinal population-based epidemiological surveys in other settings and populations, would help address these issues.

## 5. Conclusions

The present study demonstrated that hypertension had no statistically significant or clinically significant association with dental status. The study established a profile of the “patient vulnerable to hypertension”, which retained well-known risk factors for hypertension such as older age, male sex, smoking, diabetes, obesity, and fatty liver, but not dental parameters.

The present study analyzed the associations between CRP and dental conditions, and combined the statistically significant variables to create a dental inflammation score (DIS). The ROC analysis and multivariate analyses produced “fail” statistical associations between DIS and hypertension, thus the results could not support an inflammation theorem wherein dental status contributes to systemic inflammation and hypertension. The associations in the univariate analysis between hypertension and dental status may be explained by a shared common profile, and risk factors such as demographics and smoking. An appropriate risk factors management approach should be adopted by both dental and general health clinicians and health authorities, in order to control for common high-risk behaviors such as smoking, and to promote a healthy lifestyle to reduce chronic disease occurrence.

## Figures and Tables

**Table 1:** The associations between socio-demographic parameters and hypertension diagnosis among the study population and among the age-, sex- and smoking-matched sub-population.

Parameter	Variable	Hypertension (*N* = 3363) No.(%)	Comparison to Those without Hypertension among the Whole Study Population (*N* = 129,166)			Comparison to Age-, Sex- and Smoking-Matched Group without Hypertension (*N* = 10,089)		
			Without Hypertension No. (%)	*p* Value	OR (95% Confidence Interval) #	Without Hypertension No. (%)	*p* Value	OR (95% Confidence Interval) #
Sex	Men	3008 (3.0)	96,458 (97.0)	<0.001 *	2.8 (2.5–3.2)	9024 (75.0)	1.0 *	1.0 (0.8–1.1)
	Women	355 (1.1)	32,708 (98.9)		1	1065 (75.0)		1
Education	High school	1683 (1.5)	110,429 (98.5)	<0.001 ^	0.2 (0.1–0.3)	5617 (76.9)	<0.001 ^	0.9 (0.82–0.99)
	Technicians	727 (9.8)	6699 (90.2)		1.3 (1.2–1.5)	1593 (68.7)		1.3 (1.2–1.5)
	Academic	950 (7.4)	11,866 (92.6)		1	2866 (75.1)		1
Locality of residence	Urban Jewish	2960 (2.6)	110,508 (97.4)	<0.001 ^	0.3 (0.2–0.4)	8497 (74.2)	<0.001 ^	1.1 (0.8–1. 6)
	Urban non-Jewish	339 (1.9)	17,579 (98.1)		0.2 (0.1–0.3)	1372 (80.2)		0.8 (0.6–1.1)
	Rural	48 (8.2)	535 (91.8)		1	173 (77.3)		1
Socioeconomic status (SES)	Low	143 (2.5)	5576 (97.5)	<0.001 ^	1.0 (0.8–1.2)	542 (79.1)	0.012 ^	1.2 (1.1–1.6)
	Medium	1794 (2.6)	66,825 (97.4)		1.1 (1.0–1.2)	5165 (74.2)		1.06 (0.98–1.15)
	High	1357 (2.4)	55,350 (97.6)		1	4155 (75.4)		1
Rings of a city/town	Central rings	2945 (2.5)	115,505 (97.5)	0.001 *	1	9035 (75.4)	0.001 *	1
	Peripheral rings	418 (3.0)	13,661 (97.0)		1.2 (1.1–1.3)	1054 (71.6)		1.21 (1.07–1.37)
Birth country	Western Europe	486 (4.6)	10,085 (95.4)	0.001 ^	1.9 (1.8–2.2)	809 (62.5)	0.001 ^	1.9 (1.7–2.1)
	East Europe and FSU	48 (2.8)	1667 (97.2)		1.1 (0.8–1.2)	119 (71.3)		1.3 (0.9–1.8)
	Asia	27 (5.3)	482 (94.7)		2.3 (1.6–3.3)	59 (68.6)		1.5 (0.9–2.3)
	Ethiopia	29 (1.3)	2159 (98.7)		0.5 (0.4–0.8)	158 (84.5)		0.6 (0.4–0.8)
	Africa	18 (5.2)	327 (94.8)		2.2 (1.4–3.6)	45 (71.4)		1.3 (0.7–2.2)
	North America	35 (1.2)	2824 (98.8)		0.5 (0.3–0.7)	149 (81.0)		0.7 (0.52–1.09)
	South America	25 (2.6)	932 (97.4)		1.1 (0.7–1.6)	68 (73.1)		1.2 (0.7–1.8)
	Israel	2693 (2.4)	110,666 (97.6)		1	8680 (76.3)		1
Parameter	Hypertension Diagnosis		Comparison to Those without Hypertension Using the Whole Study Population (*N* = 129,166)			Comparison to Age-, Sex- and Smoking-Matched Group without Hypertension (*N* = 10,089)		
		Mean ± SD	Mean ± SD	*p* Value ^^	OR (95% Confidence Interval) **	Mean ± SD	*p* Value ^^	OR (95% Confidence Interval) **
Age (years)		29.5 ± 9.8	21.7 ± 5.7	<0.001	1.1 (1.1–1.1)	29.3 ± 9.6	1.0	1.0 (0.92–1.01)
Service duration (years)		11.1 ± 11.1	2.9 ± 5.9	<0.001	1.1 (1.1–1.1)	10.9 ± 10.7	0.93	1.0 (1.0–1.0)

* Pearson Chi-Square; ^ likelihood ratio; # binary logistic regression; ** generalized linear models; ^^ non-paired *t*-test. OR: Odds ratio; FSU: Former soviet union; SD: standard deviation.

**Table 2:** The association of hypertension diagnosis with patient health-related habits and attendance patterns among the study population and among the age-, sex- and smoking-matched sub-population.

Parameter	Variable	Hypertension (*N* = 3363) No.(%)	Comparison to Those without Hypertension among the Whole Study Population (*N* = 129,166)			Comparison to Age-, Sex- and Smoking-Matched Group without Hypertension (*N* = 10,089)		
			No Hypertension No. (%)	*p* Value *	OR (95% Confidence Interval) **	No Hypertension No. (%)	*p* Value *	OR (95% Confidence Interval) **
Brushing teeth once a day or more	No	670 (3.8)	17,150 (96.2)	<0.001	1.5 (1.4–1.7)	1932 (74.3)	0.696	0.97 (0.87–1.09)
	Yes	978 (2.5)	38,698 (97.5)		1	2885 (74.7)		1
Consumption of cariogenic diet	No	1086 (3.1)	33,435 (96.9)	<0.001	1	3281 (75.1)	0.1	1
	Yes	562 (2.4)	22,413 (97.6)		0.7 (0.6–0.8)	1537 (73.2)		1.10 (0.98–1.24)
Consumption of sweetened beverages	No	1039 (3.1)	31,970 (96.9)	<0.001	1	3126 (75.1)	0.176	1
	Yes	609 (2.5)	23,878 (97.5)		0.8 (0.7–0.9)	1691 (73.5)		1.08 (0.96–1.21)
Smoking	No	2628 (2.1)	123,017 (97.9)	<0.001	1	7884 (75.0)	1.0	1
	Yes	735 (10.7)	6149 (89.3)		5.6 (5.1–6.1)	2205 (75.0)		1.0 (0.91–1.09)
Alcohol consumption	No	3350 (2.5)	129,027 (97.5)	<0.001	1	10,070 (75.0)	0.04	1
	Yes	13 (8.6)	139 (91.4)		3.6 (2.0–6.3)	19 (59.4)		2.0 (1.0–4.1)
Parameter	Hypertension Diagnosis		Comparison to Those without Hypertension Using the Whole Study Population (*N* = 129,166)			Comparison to Age-, Sex- and Smoking-Matched Group without Hypertension (*N* = 10,089)		
	Mean ± SD		Mean ± SD	*p* Value ^	OR (95% Confidence Interval) ∨	Mean ± SD	*p* Value ^	OR (95% Confidence Interval) ∨
Total number of appointments with a general physician	19.3 ± 15.8		14.1 ± 11.8	<0.001	1.027 (1.024–1.029)	12.9 ± 11.6	<0.001	1.035 (1.032–1.038)
Total number of dental appointments	11.0 ± 16.5		5.7 ± 10.0	<0.001	1.025 (1.023–1.027)	9.4 ± 14.9	<0.001	1.006 (1.004–1.008)
Total number of non-attendance to scheduled dental appointments	1.7 ± 4.0		0.9 ± 2.6	<0.001	1.060 (1.052–1.068)	1.5 ± 3.5	0.002	1.016 (1.006–1.027)

* Pearson Chi-Square; ** binary logistic regression; ^ non-paired t-test; ∨ generalized linear models. OR: Odds ratio; SD: standard deviation.

**Table 3:** The associations between general health status and periodontal disease with hypertension diagnosis among the study population and among the age-, sex- and smoking-matched sub-population.

Parameter	Variable	Hypertension (*N* = 3363) No. (%)	Comparison to those without Hypertension among the Whole Study Population (*N* = 129,166)			Comparison to Age-, Sex- and Smoking-Matched Group without Hypertension (*N* = 10,089)		
			No Hypertension No. (%)	*p* Value *	OR (95% Confidence Interval) **	No Hypertension No. (%)	*p* Value *	OR (95% Confidence Interval) **
Hyperlipidemia	No	3235 (2.5)	128,333 (97.5)	<0.001	1	9813 (75.2)	0.002	1
	Yes	128 (13.3)	833 (86.7)		6.1 (5.0–7.3)	276 (68.3)		1.4 (1.1–1.7)
Diabetes mellitus	No	3222 (2.4)	128,962 (97.6)	<0.001	1	10,031 (75.7)	<0.001	1
	Yes	141 (40.9)	204 (59.1)		27.6 (22.2 −34.4)	58 (29.1)		7.5 (5.5–10.3)
Impaired glucose tolerance (IGT)	No	3345 (2.5)	129,056 (97.5)	<0.001	1	10,052 (75.0)	0.185	1
	Yes	18 (14.1)	110 (85.9)		6.3 (3.8–10.4)	37 (67.3)		1.4 (0.8–2.5)
Obesity	No	2058 (1.6)	123,023 (98.4)	<0.001	1	8271 (80.1)	<0.001	1
	Yes	1305 (17.5)	6143 (82.5)		12.7 (11.8–13.6)	1818 (58.2)		2.8 (2.6–3.1)
Cardiovascular disease	No	2919 (2.3)	126,012 (97.7)	<0.001	1	9460 (76.4)	<0.001	1
	Yes	444 (12.3)	3154 (87.7)		6.0 (5.4–6.7)	629 (58.6)		2.2 (2.0–2.6)
Fatty liver	No	3118 (2.4)	128,473 (97.6)	<0.001	1	9849 (76.0)	<0.001	1
	Yes	245 (26.1)	693 (73.9)		14.5 (12.5–16.9)	240 (49.5)		3.2 (2.6–3.8)
Obstructive sleep apnea (OSA)	No	3305 (2.5)	128,906 (97.5)	<0.001	1	9999 (75.2)	<0.001	1
	Yes	58 (18.2)	260 (81.8)		8.7 (6.5–11.6)	90 (60.8)		1.9 (1.4–2.7)
Anemia	No	3043 (2.4)	121,726 (97.6)	<0.001	1	9203 (75.2)	0.197	1
	Yes	320 (4.1)	7440 (95.9)		1.7 (1.5–1.9)	886 (73.5)		1.1 (0.9–1.2)
Periodontal disease	No	1431 (86.8)	50,435 (90.3)	<0.001	1	4221 (74.7)	0.401	1
	Yes	217 (13.2)	5413 (9.7)		1.4 (1.2–1.6)	596 (73.3)		1.07 (0.90–1.26)

* Pearson Chi-Square; ** binary logistic regression. OR: Odds ratio.

**Table 4:** Requirements and performance of dental procedures according to hypertension diagnosis among the study population and among the age-, sex- and smoking-matched sub-population.

Parameter	Hypertension (*N* = 3363) No. (%)	Comparison to Those without Hypertension among the Whole Study Population (*N* = 129,166)			Comparison to Age-, Sex- and Smoking-Matched Group without Hypertension (*N* = 10,089)		
		No Hypertension	*p* Value *	Hypertension vs. No Hypertension	No Hypertension	*p* Value *	Hypertension vs. No Hypertension
	Mean ± SD	Mean ± SD		OR (95% Confidence Interval) **	Mean ± SD		OR (95% Confidence Interval) **
The number of teeth that required one surface amalgam filling	0.5 ± 1.1	0.6 ± 1.2	0.019	0.9 (0.9–0.9)	0.5 ± 1.0	0.018	1.043 (1.008–1.079)
The number of teeth where one surface amalgam filling was performed	0.28 ± 0.7	0.29 ± 0.7	0.537	0.9 (0.9–1.0)	0.27 ± 0.7	0.350	1.025 (0.973–1.079)
The number of teeth that required two amalgam fillings on two surfaces	1.8 ± 1.3	2.0 ± 1.4	<0.001	0.9 (0.8–0.9)	1.8 ± 1.3	0.692	1.011 (0.958–1.067)
The number of teeth where two surface amalgam fillings were performed	0.39 ± 0.85	0.32 ± 0.8	<0.001	1.085 (1.047–1.125)	0.37 ± 0.83	0.247	1.027 (0.981–1.075)
The number of teeth that required three and more amalgam fillings on surfaces	0.13 ± 0.5	0.11 ± 0.4	<0.001	1.111 (1.040–1.185)	0.12 ± 0.4	0.085	1.079 (0.994–1.171)
The number of teeth where three and more surface amalgam fillings were performed	0.09 ± 0.3	0.07 ± 0.3	<0.001	1.205 (1.106–1.314)	0.10 ± 0.37	0.580	0.970 (0.871–1.081)
The number of teeth that required amalgam crowns	0.02 ± 0.1	0.01 ± 0.1	0.001	1.588 (1.289–1.957)	0.02 ± 0.1	0.839	1.072 (0.932–1.233)
The number of teeth where amalgam crowns were performed	0.06 ± 0.3	0.03 ± 0.2	<0.001	1.720 (1.528–1.937)	0.05 ± 0.2	0.175	0.925 (0.827–1.034)
The number of teeth that required resin-based composite fillings on one to four surfaces, anterior	0.27 ± 0.8	0.24 ± 0.8	0.048	1.042 (1.001–1.083)	0.26 ± 0.8	0.446	1.019 (0.971–1.069)
The number of teeth where resin-based composite fillings were performed on one to four surfaces, anterior	0.34 ± 1.3	0.25 ± 0.8	<0.001	1.077 (1.049–1.107)	0.31 ± 1.07	0.270	1.018 (0.986–1.051)
Total number of teeth that required fillings	1.57 ± 2.3	1.55 ± 2.4	0.010	1.003 (0.989–1.017)	1.46 ± 2.2	0.016	1.021 (1.004–1.039)
Total number of teeth where fillings were performed	1.2 ± 2.1	1.0 ± 1.9	<0.001	1.045 (1.030–1.060)	1.15 ± 2.04	0.222	1.011 (0.993–1.030)
Total number of teeth that required endodontic treatment	0.12 ± 0.4	0.08 ± 0.3	<0.001	1.199 (1.124–1.278)	0.10 ± 0.4	0.025	1.113 (1.017–1.219)
Total number of teeth where endodontic treatment was performed	0.14 ± 0.4	0.07 ± 0.3	<0.001	1.421 (1.329–1.520)	0.12 ± 0.4	0.020	1.112 (1.019–1.214)
The number of teeth that required prefabricated (direct, post and core)	0.15 ± 0.5	0.10 ± 0.4	<0.001	1.211 (1.140–1.285)	0.13 ± 0.4	0.173	1.058 (0.980–1.142)
The number of teeth on which prefabricated (direct, post and core) was performed	0.19 ± 0.7	0.09 ± 0.3	<0.001	1.398 (1.327–1.473)	0.15 ± 0.5	0.004	1.117 (1.048–1.191)
The number of teeth that required crowns	0.22 ± 0.7	0.15 ± 0.5	<0.001	1.165 (1.115–1.217)	0.19 ± 0.6	0.074	1.053 (0.997–1.112)
The number of teeth where a crown was performed	0.19 ± 0.13	0.05 ± 0.52	<0.001	1.180 (1.146–1.215)	0.17 ± 0.97	0.244	0.991 (0.585–1.678)
The number of teeth that required extractions	0.20 ± 0.6	0.14 ± 0.5	<0.001	1.187 (1.129–1.248)	0.18 ± 0.6	0.098	1.053 (0.991–1.119)
Total number of teeth where extractions were performed	0.18 ± 0.6	0.09 ± 0.4	<0.001	1.253 (1.193–1.316)	0.14 ± 0.6	0.004	1.084 (1.023–1.150)
Missing teeth	1.05 ± 1.72	0.57 ± 1.27	<0.001	1.150 (1.124–1.316)	0.94 ± 1.55	0.017	1.043 (1.009–1.078)

* non-paired *t* test, ** generalized linear models.

**Table 5:** Multivariate logistic regression analysis for hypertension diagnosis as the dependent variable with statistically significant independent parameters among the whole study population (*N* = 132,529).

Parameter	B	Standard Error	*p* Value	Exp(B) and 95% Confidence Interval for Exp (B)	Collinearity Statistics *	
					Tolerance	VIF
(Intercept)	−0.6	0.6	0.281			
Age (years)	0.05	0.04	<0.001	1.05 (1.04–1.06)	0.541	1.850
Sex: men vs. women	0.6	0.08	<0.001	1.9 (1.6–2.2)	0.924	1.082
Smoking	1.8	0.07	0.016	1.2 (1.05–1.4)	0.755	1.325
Locality of residence: Urban non-Jewish vs. Urban Jewish	0.3	0.5	0.489	1.4 (0.5–3.6)	0.983	1.017
Urban Jewish—Rural vs. Urban Jewish	0.0	0.5	1.000	1.0 (0.3–2.6)	0.989	1.011
Socio-economic status (SES)—low vs. high	−0.2	0.1	0.126	0.8 (0.6–1.0)	0.946	1.057
Socio-economic status (SES)—medium vs. high	−0.1	0.05	0.068	0.9 (0.8–1.0)	0.949	1.054
Birth country: Western Europe vs. native Israeli	0.6	0.07	<0.001	1.9 (1.6–2.2)	0.988	1.012
Birth country: East Europe and FSU vs. native Israeli	0.5	0.3	0.098	1.6 (0.9–3.0)	0.997	1.003
Birth country: Asia vs. native Israeli	0.4	0.2	0.024	1.6 (1.1–2.3)	0.992	1.008
Birth country: Ethiopia vs. native Israeli	−0.3	0.2	0.194	0.7 (0.4–1.2)	0.991	1.009
Birth country: Africa vs. native Israeli	−0.8	0.5	0.104	0.4 (0.1–1.1)	0.997	1.003
Birth country: North America vs. native Israeli	0.1	0.2	0.612	1.1 (0.7–1.7)	0.992	1.008
Birth country: South America vs. native Israeli	0.4	0.2	0.105	1.5 (0.9–2.6)	0.998	1.002
Total number of teeth that required fillings	0.02	0.01	0.094	1.02 (0.9–1.04)	0.921	1.086
Total number of teeth that required endodontic treatment	0.001	0.06	0.990	1.0 (0.8–1.1)	0.931	1.075
Missing teeth	0.02	0.01	0.236	1.0 (0.9–1.0)	0.927	1.079
Periodontal disease	0.04	0.08	0.600	1.0 (0.8–1.2)	0.985	1.015
The number of teeth that required crowns	0.004	0.06	0.955	1.0 (0.8–1.1)	0.978	1.022
Hyperlipidemia	0.2	0.1	0.178	1.2 (0.9–1.6)	0.957	1.045
Diabetes mellitus	1.4	0.1	<0.001	4.0 (2.9–5.7)	0.962	1.039
Obesity	1.4	0.07	<0.001	4.2 (3.7–4.9)	0.698	1.432
Cardiovascular disease	0.6	0.1	<0.001	1.9 (1.6–2.3)	0.934	1.070
Fatty liver	0.6	0.1	<0.001	1.8 (1.5–2.3)	0.909	1.100
Obstructive sleep apnea	0.2	0.2	0.279	1.2 (0.8–1.8)	0.973	1.028
Anemia	0.1	0.09	0.257	1.1 (0.9–1.3)	0.919	1.088

* Collinearity statistics: linear regression analysis. VIF: variance inflation factor.

**Table 6:** Multivariate logistic regression analysis for hypertension diagnosis as the dependent variable with statistically significant independent parameters among the sub-population of age-, sex- and smoking-matched subjects.

Parameter	B	Standard Error	*p* Value	Exp(B) and 95% Wald Confidence Interval for Exp (B)	Collinearity Statistics *	
					Tolerance	VIF
(Intercept)	**1.6**	**0.6**	**0.01**			
Locality of residence—Urban non-Jewish vs. Urban Jewish	0.5	0.5	0.265	1.7 (0.6–4.7)	0.977	1.024
Urban Jewish—Rural vs. Urban Jewish	0.2	0.5	0.633	1.2 (0.4–3.5)	0.988	1.012
Socio-economic status (SES)—low vs. high	0.3	0.1	0.026	1.4 (1.03–1.8)	0.947	1.056
Socio-economic status (SES)—medium vs. high	0.1	0.06	0.023	1.1 (1.02–1.3)	0.915	1.093
Birth country: Western Europe vs. native Israeli	0.6	0.09	<0.001	1.9 (1.6–2.3)	0.642	1.558
Birth country: East Europe and FSU vs. native Israeli	0.4	0.2	0.063	1.5 (0.9–2.5)	0.832	1.202
Birth country: Asia vs. native Israeli	0.5	0.3	0.103	1.7 (0.8–3.4)	0.936	1.068
Birth country: Ethiopia vs. native Israeli	−0.3	0.3	0.225	0.7 (0.3–1.2)	0.925	1.081
Birth country: Africa vs. native Israeli	−0.4	0.4	0.352	0.6 (0.2–1.6)	0.925	1.081
Birth country: North America vs. native Israeli	0.3	0.2	0.253	1.3 (0.8–2.3)	0.842	1.187
Birth country: South America vs. native Israeli	0.5	0.3	0.077	1.7 (0.9–3.3)	0. 898	1.114
Education: high school vs. academic	0.4	0.08	<0.001	1.5 (1.3–1.8)	0.745	1.343
Education: technician vs. academic	0.1	0.09	0.228	1.7 (0.9–3.3)	0.751	1.332
Total number of teeth that required fillings	0.02	0.01	0.059	1.02 (0.9–1.0)	0.930	1.075
Total number of teeth that required endodontic treatment	0.03	0.09	0.700	1.03 (0.8–1.2)	0.943	1.060
Missing teeth	0.005	0.02	0.800	0.9 (0.9–1.0)	0.932	1.073
Hyperlipidemia	0.04	0.1	0.809	1.08 (0.7–1.4)	0.974	1.027
Diabetes mellitus	1.4	0.2	<0.001	4.08 (2.6–6.1)	0.949	1.054
Obesity	1.0	0.07	<0.001	2.7 (2.4–3.2)	0.770	1.298
Cardiovascular disease	0.6	0.09	<0.001	1.8 (1.5–2.2)	0.939	1.065
Fatty liver	0.5	0.1	<0.001	1.7 (1.3–2.3)	0.910	1.099
Obstructive sleep apnea	0.2	0.2	0.265	1.3 (0.8–2.02)	0.974	1.026

* Collinearity statistics: linear regression analysis. VIF: variance inflation factor.

**Table 7:** General linear model with C-reactive protein (CRP) as a dependent variable against the statistically significant dental parameters and the establishment of appropriate weights for the dental inflammation score (DIS).

	OR 95% CI	*p* Value	Omnibus Test *		Likelihood Ratio of Each Variable Out of Total × 100
			Likelihood Ratio Chi Square	*p* Value	
Periodontal disease	1.179 (1.044–1.331)	0.008	6.77	0.009	(6.77/48.93) × 100 = 13.84≈14
The number of teeth that required crowns	1.160 (1.028–1.308)	0.016	5.24	0.022	(5.24/48.93) × 100 = 10.71≈11
Missing teeth	1.081 (1.056–1.106)	<0.001	36.91	<0.001	(36.91/48.93) × 100 = 75.44≈75
Total			Total = 48.93		

* Omnibus test—Compare the fitted model against the intercept-only model. CI: Confidence Interval.

## Data Availability

Data sharing not applicable.

## Abbreviations

BMI	Body mass index
MPR	Medical patient record
CRP	C-reactive protein
CVD	Cardiovascular disease
DMF	Decayed, missed, filled teeth
DPR	Dental patient record
HDL	High-density lipoprotein
IDF	Israeli Defense Force
MetS	Metabolic syndrome
OSA	Obstructive sleep apnea
PD	Periodontal disease
SES	Socio-economic status
TIA	Transient ischemic attack
VLDL	Very low-density lipoprotein

## Appendix

**Table A1:** Age-, sex- and smoking-matched strata according to hypertension (likelihood ratio: *p* = 1.0).

Strata	Hypertension Diagnosis		Total *N* (%)
	No *N* (%)	Yes *N* (%)	
Female younger non-smoker	699 (75)	233 (25)	932 (100)
Female older non-smoker	204 (75)	68 (25)	272 (100)
Female younger smoker	51 (75)	17 (25)	68 (100)
Female older smoker	111 (75)	37 (25)	148(100)
Male younger non-smoker	4461 (75)	1487 (25)	5948 (100)
Male older non-smoker	2520 (75)	840 (25)	3360 (100)
Male younger smoker	297 (75)	99 (25)	396 (100)
Male older smoker	1746 (75)	582 (25)	2328 (100)
Total	10,089 (75)	3363 (25)	13,452 (100)
